# The endocannabinoid anandamide during lactation increases body fat content and CB_1_ receptor levels in mice adipose tissue

**DOI:** 10.1038/nutd.2015.17

**Published:** 2015-06-22

**Authors:** C A Aguirre, V A Castillo, M N Llanos

**Affiliations:** 1 Instituto de Nutrición y Tecnología de los Alimentos (INTA), Universidad de Chile, Santiago, Chile

## Abstract

Type 1 cannabinoid receptors (CB_1_R) modulate energy balance; thus, their premature activation may result in altered physiology of tissues involved in such a function. Activation of CB_1_R mainly occurs after binding to the endocannabinoid Anandamide (AEA). The objective of this study was to evaluate the effects of AEA treatment during lactation on epididymal and body fat content, in addition to CB_1_R protein level at weaning. With this purpose, male mice pups were orally treated with AEA (20 μg g^−1^ body weight) or vehicle during lactation. Mice (21 days old) were killed and epididymal fat was extracted to evaluate its amount, adipocyte size and CB_1_R protein levels by western blot analysis. Total body fat percentage was also evaluated. Anandamide-treated mice showed an increased body fat content at 21 and 150 days of age. Moreover, epididymal adipose tissue amount, adipocyte size and CB_1_R protein levels were higher in the AEA-treated group. This *in vivo* study shows for the first time that a progressive increase in body fat accumulation can be programmed in early stages of life by oral treatment with the endocannabinoid AEA, a fact associated with an increased amount of epididymal fat pads and a higher expression of CB_1_R in this tissue.

## Introduction

Present evidence from epidemiological and animal studies indicates that events occurring during fetal and/or neonatal life could have long-term health consequences.^1^ Stress constitutes an early stimulus leading to overweight and metabolic alterations in adulthood associated with a perturbed endocannabinoid system (ECS), as previously reported.^2^

The ECS consists of Type 1 and 2 cannabinoid (CB_1_, CB_2_) receptors, which are present in several tissues including the central nervous system, adipose tissue, liver and pancreas.^3, 4, 5^ Their endogenous ligands are known as endocannabinoids, with arachidonoylethanolamide or anandamide (AEA) and 2-arachidonoyl-glycerol (2-AG) being the most studied agonists. These endocannabinoids have endocrine, autocrine and paracrine actions.^6, 7^ Stress elevates endocannabinoid levels in some areas of the central nervous system and activates CB_1_R involved in the negative feedback mechanism to repress the activity of the hypothalamus–pituitary–adrenal axis.^8^ In addition, activation of CB_1_R in some peripheral tissues has been related to overweight/obesity, insulin and leptin resistance and dyslipidemia.^9, 10^ We have previously demonstrated that oral administration of the AEA during lactation results in overweight, epididymal fat accumulation, hormonal disruption and a marked state of insulin resistance in adult mice. Moreover, a higher expression of CB_1_R in epididymal fat of adult mice was also observed.^11^

Adipocytes not only express CB_1_R (a target for AEA), its activation is involved in adipocyte growth and differentiation, modulation of adipokine secretion and stimulation of lipogenesis.^9, 12, 13^ Nevertheless, these studies have been performed in cultured adipocytes, and there is no information whether some of these effects may be observed in *in vivo* experiments.

With all these antecedents in mind, the aim of this study was to evaluate the *in vivo* effects of oral administration of anandamide during lactation on epididymal adipose tissue development and total body fat content, and whether these factors were associated with increased amounts of CB_1_R immediately after weaning.

## Materials and methods

Bioethics Committee for Animal Experimentation of the Institute of Nutrition and Food Technology, University of Chile, Santiago approved this study.

### Animals

Synchronously primiparous pregnant Swiss CD-1 mice were housed under standardized conditions of humidity, temperature (22–24 °C) and a 12:12-h light–dark cycle. Animals had free access to purified deionized tap water and a standard diet.^11^

From day 15, pregnant female mice were examined twice a day for the presence of pups. Newborn pups, 6–8 litters of homogeneous size (12–14 pups), were put together and male pups were separated from female pups. Subsequently, six male pups having homogeneous weights were randomly selected and assigned to a substitute mother for random cross-lactation. This procedure is used to homogenize genetic and behavioral factors affecting metabolic characteristics. Next, animals were distributed to constitute the following groups.

Control mice: from days 1 to 21 of lactation, pups were daily weighed and treated with an oral dose of soy oil (1 μl g^−1^ of body weight) by means of an automatic pipette with an appropriate tip.
AEA-treated mice: from days 1 to 21 of lactation, pups were daily weighed and treated with an oral dose of a solution of AEA 20 mg ml^−1^ in soy oil (20 μg g^−1^ body weight; Sigma Chem Co, St Louis, MO, USA).

After weaning, mice were killed^14^ and epididymal fat pads were extracted, weighed and quickly frozen until analysis.

#### Body fat content

Body fat content was determined by acid hydrolysis. After death, animals were frozen, pulverized and a 5g aliquot of each mouse was homogenized with 20 ml of 6 N hydrochloric acid. The sample was subsequently heated and shaken during 45 min at 80 °C. Next, 1 g of celite was added and finally filtered under vacuum. The filter was dried at 103±2 °C for 1 h, and fat previously absorbed in celite was extracted and quantified. This method is similar to that used to validate magnetic resonance spectroscopy for evaluating body composition.^15^

#### Western blot analysis of CB_1_R

Epididymal fat (100 mg) was homogenized in 500 μl of RIPA buffer (25 mM Tris-HCl, pH7.6, 150 mM NaCl, 1% sodium deoxycholate, 0.1% SDS) in the presence of a protease inhibitor cocktail (Sigma Chem Co, P2714). Proteins were separated in a 10% SDS-polyacrylamide gel and subsequently transferred to a polyvinylidene fluoride membrane at 4 °C. Rabbit polyclonal antibody for CB_1_R was used as the primary antibody (Cayman Chemical, Irvine, CA, USA), and an enzyme-conjugated anti-rabbit antibody was the secondary antibody (Bio-Rad, Ann Arbor, CA, USA). Appropriate positive and negative controls with brain tissue and the corresponding CB_1_R antibody blocking peptide, respectively, were also performed (Cayman Chemical).

#### Adipocyte size

Epididymal fat pads were washed in warm saline and fixed in 4% Böuin solution for 48 h, and then included in solid paraffin and sliced in 10-μm-thick sections, which were mounted onto gelatin-coated glass slides. The morphometric analysis (estimated mean volume) was carried out under light microscopy. Six slices for each animal and three randomly selected fields per slice were considered.

### Statistical analysis

Data are expressed as mean±s.e.m. Shapiro–Wilk's and Levene tests were carried out to evaluate normal distribution and variance homogeneity. When appropriate, the Mann–Whitney *U*-Test statistical analysis was performed. Significance was set at *P*⩽0.05. All analyses were conducted using Stata 10.1 statistical package (StataCorp LP, College Station, TX, USA).

## Results

### Body and epididymal fat contents

**Figure 1:**
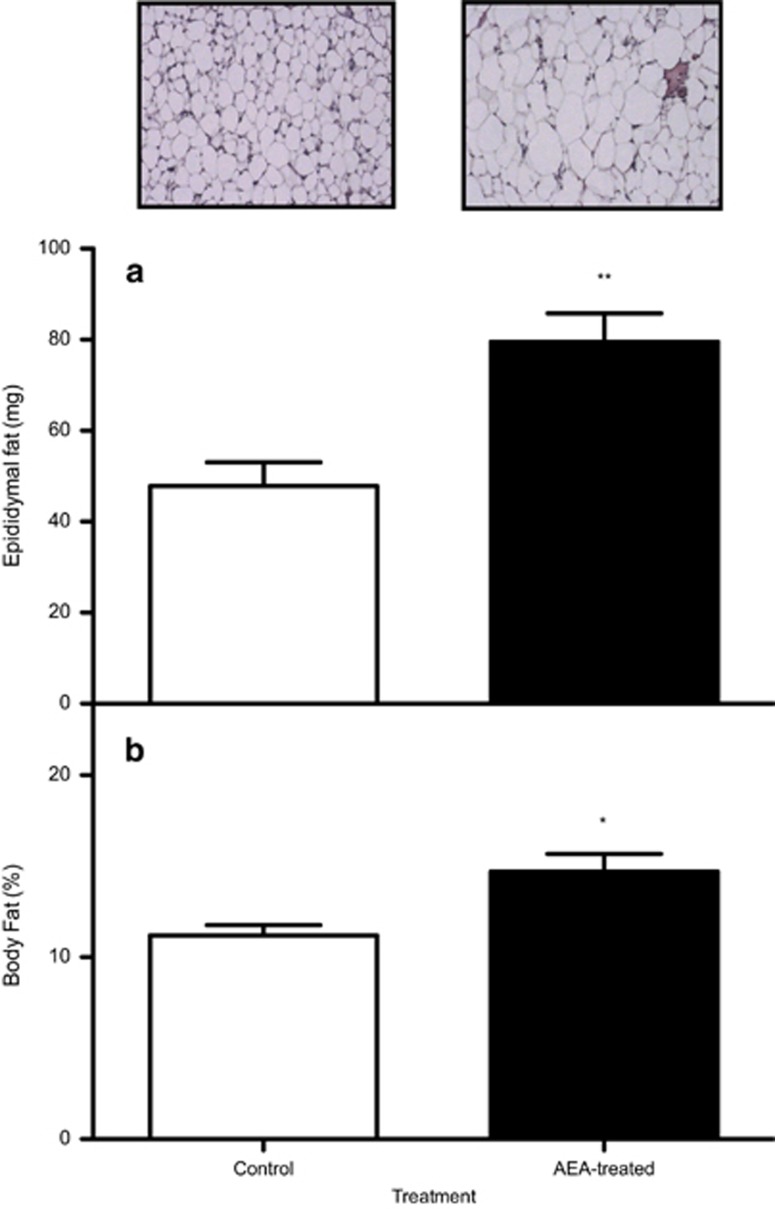
(**a**) Effect of AEA treatment during lactation on the total amount of epididymal fat and adipocyte size in 21-day-old control and AEA-treated animals. **P<0.01 (Mann–Whitney *U*-test; mean±s.e.m.; *n*=6 animals/group). (**b**) Effect of AEA treatment during lactation on total body fat content in 21-day-old mice. *P<0.05 (Mann–Whitney *U*-test; mean±s.e.m.; *n*=6 animals/group).

Figure 1a shows that 21-day-old AEA-treated mice had significantly higher epididymal fat content compared with control animals together with higher adipocyte size (12.1±0.9 × 10^3^ vs 6.8±0.3 × 10^3^ μm^3^, respectively; *P*<0.05). Body fat content was determined in 21- and 150-day-old AEA-treated and control animals. Our previous results have demonstrated that AEA-treated, 21-day-old animals had no significant differences on body weight in comparison with control mice.^11^ Notwithstanding, body fat content was 31% higher in the AEA-treated mice (11.2±0.6% vs 14.7±1.0% mean±s.e.m.; *n*=6; *P*<0.05; Figure 1b). The accumulation of body fat was a remarkable progressive process during life span of AEA-treated mice during lactation, being 322% higher in 150-day-old AEA-treated animals than in control animals (28.8±0.5% vs 8.9±1.3%, respectively). This fat accumulation was reflected in the body weight of AEA-treated animals, due only in part, to a higher accumulative food intake after weaning.^11^

### Western blot of CB_1_R in epididymal fat

**Figure 2:**
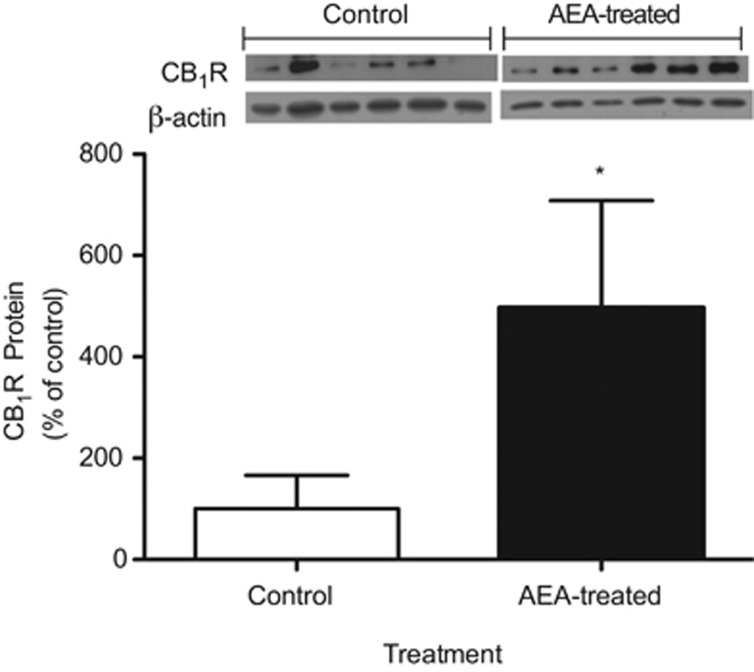
Western blot analysis of relative levels of CB_1_R in epididymal fat of AEA-treated and control mice at 21 days. For densitometric quantification purposes, β-actin was used as a loading control. Results are expressed as percentage relative to the CB_1_R expression in control mice. *P<0.05 (Mann–Whitney *U*-test; mean±s.e.m.; *n*=6).

Figure 2 shows that CB_1_R protein in epididymal fat from 21-day-old AEA-treated mice was significantly higher than levels found in control animals. It is worth noting that epididymal fat amount was coincident with the CB_1_R protein level found in these animals.

## Discussion

This *in vivo* study shows for the first time that mice treated with anandamide during lactation show increased epididymal and total body fat content, in addition to larger adipocytes in epididymal adipose tissue concomitant to higher levels of CB_1_R at weaning. In a previous report, it has been shown that adult female mice treated with a low dose of AEA (0.001 mg kg^−1^) increased food intake.^16^ In this way, it may be argued that our lactating animals may program long-term body weight and fat content owing to a higher consumption of maternal milk during lactation. However, as previously stated, the effect of AEA on appetite may be variable and even absent depending on the dose and experimental design.^16^ As our AEA-treated animals did not show any difference in body weight in comparison with control animals during lactation, our results indicate that effects of early AEA treatment on long-term adult body weight and fat content are likely owing to an increased amount and/or overactivity of CB_1_R in visceral adipose tissue.^11^

Several *in vitro* studies have previously shown that the endocannabinoid system is involved in adipogenesis and lipogenesis of the adipose tissue. It has been shown that AEA increased the expression and activation of PPAR-γ, leading to differentiation of fibroblasts to adipocytes.^13^ Moreover, CB_1_R activation with the synthetic agonist WIN-55.212-2 stimulated several enzymes associated with intracellular lipid accumulation,^17^ including augmented lipoprotein lipase activity in cultured adipocytes.^9^ If we consider that administration of AEA during lactation increased CB_1_R levels in adipose tissue in 21-day-old mice, which are maintained until adulthood, this condition could be involved in the progressive fat accumulation observed from 21- until 150-day-old AEA-treated animals, likely involved in insulin resistance and dyslipidemia, as we have previously shown.^11^

Diet may be a factor involved in elevated levels of endocannabinoids in different tissues. In fact, it has been recently shown that increasing dietary linoleic acid (LNA) elevates arachidonic acid and therefore endocannabinoid levels and adiposity in mice.^18^ In humans, an epidemiological report has linked increased intake of LNA over time to increased prevalence of obesity and postulated that arachidonic acid-induced elevation of the endocannabinoid 2-AG may have altered the energy balance.^19^

If we consider that human milk contains endocannabinoids,^20^ mothers with a higher intake of LNA could enrich their milk with endocannabinoids, in this way having a chance to induce an early programming effect on body fat accumulation during life span.

Early availability of endocannabinoids and its suggested involvement in programming body fat development, leading to unhealthy conditions during adulthood, guarantee further research in this area.

## References

[bib1] Barker DJ Osmond C Diet and coronary heart disease in England and Wales during and after the second world war J Epidemiol Community Health 1986 40 37 44 3711768 10.1136/jech.40.1.37 PMC1052486

[bib2] Valenzuela CA Castillo VA Aguirre CA Ronco AM Llanos MN The CB_1_ receptor antagonist SR141716A reverses adult male mice overweight and metabolic alterations induced by early stress Obesity (Silver Spring) 2011 19 29 35 20559305 10.1038/oby.2010.131

[bib3] Cota D Marsicano G Lutz B Vicennati V Stalla GK Pasquali R Endogenous cannabinoid system as a modulator of food intake Int J Obes Relat Metab Disord 2003 27 289 301 12629555 10.1038/sj.ijo.0802250

[bib4] Bensaid M Gary-Bobo M Esclangon A Maffrad JO Le Fur G Oury-Donat F The cannabinoid CB_1_ receptor antagonist SR141716 increases Acrp30 mRNA expression in adipose tissue of obese fa/fa rats and in cultured adipocyte cells Mol Pharmacol 2003 63 908 914 12644592 10.1124/mol.63.4.908

[bib5] Osei-Hyiaman D DePetrillo M Pacher P Liu J Radaeva S Bátkai S Endocannabinoid activation at hepatic CB_1_ receptors stimulates fatty acid synthesis and contributes to diet-induced obesity J Clin Invest 2005 115 1298 1305 15864349 10.1172/JCI23057 PMC1087161

[bib6] Maccarrone M Dainese E Oddi S Intracellular trafficking of anandamide: new concepts for signaling Trends Biochem Sci 2010 35 601 608 20570522 10.1016/j.tibs.2010.05.008

[bib7] Côté M Matias I Lemieux I Petrosino S Alméras N Després JP Circulating endocannabinoid levels, abdominal adiposity and related cardiometabolic risk factors in obese men Int J Obes (Lond) 2007 31 692 699 17224929 10.1038/sj.ijo.0803539

[bib8] Hill MN Patel S Campolongo P Tasker JG Wotjak CT Bains JS Functional interactions between stress and the endocannabinoid system: from synaptic signaling to behavioral output J Neurosci 2010 30 14980 14986 21068301 10.1523/JNEUROSCI.4283-10.2010 PMC3073528

[bib9] Cota D Marsicano G Tschöp M Grübler Y Flachskamm C Schubert M The endogenous cannabinoid system affects energy balance via central orexigenic drive and peripheral lipogenesis J Clin Invest 2003 112 423 431 12897210 10.1172/JCI17725 PMC166293

[bib10] Liu J Zhou L Xiong K Godlewski G Mukhopadhyay B Tam J Hepatic cannabinoid receptor-1 mediates diet-induced insulin resistance via inhibition of insulin signaling and clearance in mice Gastroenterology 2012 142 e1 10.1053/j.gastro.2012.01.032 PMC3482511 22307032

[bib11] Aguirre CA Castillo VA Llanos MN Excess of the endocannabinoid anandamide during lactation induces overweight, fat accumulation and insulin resistance in adult mice Diabetol Metab Syndr 2012 4 35 22823902 10.1186/1758-5996-4-35 PMC3439322

[bib12] Bellocchio L Cervino C Vicennati V Pasquali R Pagotto U Cannabinoid type 1 receptor: another arrow in the adipocytes' bow J Neuroendocrinol 2008 20 130 138 18426512 10.1111/j.1365-2826.2008.01682.x

[bib13] Bouaboula M Hilairet S Marchand J Fajas L Le Fur G Casellas P Anandamide induced PPARgamma transcriptional activation and 3T3-L1 preadipocyte differentiation Eur J Pharmacol 2005 517 174 181 15987634 10.1016/j.ejphar.2005.05.032

[bib14] Euthanasia R of the A panel on No Title J Am Vet Med Assoc 2001 218 669 696 11280396 10.2460/javma.2001.218.669

[bib15] Mystkowski P Shanklend E Schreyer SA LeBoeuf RC Schwartz RS Cummings DE Validation of whole-body magnetic resonance spectroscopy as a tool to assess murine body composition Int J Obes 2000 24 719 724 10.1038/sj.ijo.0801231 10878678

[bib16] Hao S Avraham Y Mechoulam Berry E Low dose anandamide affects food intake, cognitive function, neurotransmitter and corticosterone levels in diet-restricted mice Eur J Pharmacol 2000 392 147 156 10762668 10.1016/s0014-2999(00)00059-5

[bib17] Matias I Gonthier MP Orlando P Martiadis V De Petrocellis L Carevino C Regulation, function, and dysregulation of endocannabinoids in models of adipose and beta-pancreatic cells and in obesity and hyperglycemia J Clin Endocrinol Metab 2006 91 3171 3180 16684820 10.1210/jc.2005-2679

[bib18] Alvheim AR Torstensen BE Lin YH Lillefosse HH Lock EJ Madsen L Dietary linolenic acid elevates the endocannabinoids 2-AG and anandamide and promotes weight gain in mice fed a low fat diet Lipids 2014 49 59 69 24081493 10.1007/s11745-013-3842-y PMC3889814

[bib19] Ailhaud G Massiera F Weill P Legrand P Alessandri J-M Guesnet P Temporal changes in dietary fats: role of n-6 polyunsaturated fatty acids in excessive adipose tissue development and relationship to obesity Prog Lipid Res 2006 45 203 236 16516300 10.1016/j.plipres.2006.01.003

[bib20] Marczylo TH Lam PMW Nallendran V Taylor AH Konje JC A solid-phase method for the extraction and measurement of anandamide from multiple human biomatrices Anal Biochem 2009 384 106 113 18823934 10.1016/j.ab.2008.08.040

